# Central nervous system involvement in Waldenström macroglobulinemia: a comparative population-based study of Bing-Neel syndrome and histological transformation

**DOI:** 10.1007/s00277-025-06194-4

**Published:** 2025-01-24

**Authors:** Simon Østergaard, Lars Munksgaard, Troels Hammer, Torsten Holm Nielsen, Mette Ølgod Pedersen, Lise Mette Rahbek Gjerdrum

**Affiliations:** 1 Department of Pathology, Copenhagen University Hospital - Zealand University Hospital Roskilde, Roskilde, Denmark; 2 Department of Clinical Medicine, University of Copenhagen, Copenhagen, Denmark; 3 Department of Hematology, Zealand University Hospital Roskilde, Roskilde, Denmark; 4 Department of Hematology, Copenhagen University Hospital - Rigshospitalet, Copenhagen, Denmark; 5 Danish Medicines Agency, Copenhagen, Denmark

**Keywords:** Waldenström macroglobulinemia, Bing Neel syndrome, Central nervous system, Histological transformation

## Abstract

Central nervous system (CNS) involvement in Waldenström macroglobulinemia (WM) is a rare complication that can manifest as Bing-Neel syndrome (BNS) or as histological transformation (HT) to diffuse large B-cell lymphoma (DLBCL). We report data from a single-center cohort of 469 patients consecutively diagnosed with WM between 2000 and 2022. BNS was identified in 1.5% (*n* = 7) and HT with CNS involvement (CNS-HT) in 1.7% (*n* = 8) of patients. The cumulative incidence of BNS and CNS-HT at 15 years was 2.6% and 2.7%, respectively, with CNS-HT more likely to develop in closer proximity to the initial WM diagnosis. One patient with CNS-HT exhibited a preceding phase of BNS before transformation. In general, patients with BNS and CNS-HT presented with diverse neurological symptoms and clinical features. Parenchymal lesions were uniformly found in all patients with CNS-HT, while neuroimaging findings were less consistent in patients with BNS. Involvement of multiple extramedullary sites was observed in approximately half of the patients with both BNS and CNS-HT. Patients with CNS-HT had poor outcomes, with a median overall survival of 10 months following the onset of CNS involvement, whereas BNS was associated with a more favorable prognosis, particularly in patients treated with ibrutinib. This study is the first to present a comparative analysis of BNS and CNS-HT in WM, providing novel insights into their incidence, clinical features, and outcomes.

## Introduction

Waldenström macroglobulinemia (WM) is a B-cell malignancy characterized by infiltration of a lymphoplasmacytic lymphoma (LPL) in the bone marrow (BM) and an IgM M component [1]. Some patients with WM also develop extramedullary disease, which can affect the central nervous system (CNS), manifesting as either Bing-Neel syndrome (BNS) or histological transformation (HT) to diffuse large B-cell lymphoma (DLBCL) [2–4].

BNS refers to the infiltration of malignant lymphoplasmacytic cells into the CNS [5, 6]. It is a rare complication, primarily reported in retrospective studies, with an estimated incidence of 1–5% in patients with WM [6–8]. BNS presents with a wide range of clinical symptoms, none of which are pathognomonic, leading to variable outcomes in previous reports [8–10]. Due to its rarity and clinical heterogeneity, along with a potential to occur without concurrent systemic progression, early detection can be challenging often resulting in a delayed diagnosis [6].

HT to DLBCL in WM patients has been reported with a cumulative incidence of 1% at 5 years and 3.8% at 15 years post-diagnosis [11]. CNS involvement is a frequent complication in patients with transformed WM affecting up to 23% of cases [12]. In general, patients with HT have a poor prognosis, with a median survival ranging from 16 to 32 months [4, 11]. This is further reflected in patients with CNS-HT, where one study found a median survival of 1 year after CNS involvement [12].

While patients with CNS involvement may present with similar symptoms and clinical features, BNS and CNS-HT differ markedly in terms of required treatment strategies and outcomes. This emphasizes the need for comparative studies to better discriminate between the two conditions. We present data from a population-based cohort of WM patients providing novel insights into the incidence, clinical characteristics, and outcomes of patients with WM and CNS involvement.

## Methods

### Study population

In this retrospective cohort study, we identified all patients diagnosed with Waldenström macroglobulinemia (WM) between 2000 and 2022 in Region Zealand, Denmark, which serves a population of 850,000. Patients were identified using the regional electronic healthcare database (EpicCare), and only patients diagnosed with WM according to consensus guidelines were included [13, 14]. Pathology reports of patients with CNS involvement were reviewed by an expert hematopathologist. The diagnosis of BNS was defined by the presence of LPL cells in the cerebrospinal fluid (CSF) or in a brain biopsy. HT to DLBCL with CNS involvement was defined as the presence of DLBCL in the CSF or in a brain biopsy, with a prior or concomitant diagnosis of WM. Patients with magnetic resonance imaging (MRI) or computed tomography (CT) findings clearly indicating CNS involvement, along with a simultaneous biopsy from any extramedullary site confirming DLBCL, were also considered as HT with CNS involvement. The study was approved by the Danish National Research Ethics Committee (ID number 2113049) and the Region Zealand Data Protection Agency (ID number REG-020-2022).

### Clinical data

Clinical data were collected through review of medical records. Information was recorded at the time of WM diagnosis and the onset of CNS involvement. Survival data were obtained during the last follow-up in December 2023. Treatment data were collected for first-line therapy of WM, BNS, or DLBCL, as well as the number of WM-directed therapies prior to the CNS event. Symptoms associated with CNS involvement were classified as balance disorders, cranial nerve involvement, cognitive impairment, paresis and motor/sensory symptoms, headache, cauda equina syndrome, visual disturbances, hearing deficits, or psychiatric symptoms. CNS disease locations were divided into parenchymal (including intraocular) and leptomeningeal involvement.

### Molecular data

The results of routine diagnostic testing for the MYD88 L265P mutation were retrospectively collected for patients with available molecular data. In patients with CNS involvement and unknown MYD88 L265P mutational status, the mutation was assessed by quantitative PCR (qPCR) using the qBiomarker somatic mutation PCR assay, provided that adequate formalin-fixed paraffin-embedded tissue samples were available. Tissue samples analyzed included BM from the initial WM diagnosis and brain parenchyma biopsies obtained at the time of CNS involvement. Data from fluorescence in situ hybridization (FISH) analysis of diagnostic DLBCL samples were collected to identify double-hit translocations involving MYC, BCL2, and/or BCL6. The cell of origin (COO), determined by immunohistochemistry using the Hans et al. algorithm, was also collected and categorized as either germinal center (GC) or non-GC phenotypes [15].

### Statistics

Continuous variables were presented as median values with ranges or 95% confidence intervals (CI), while categorical variables were summarized as frequencies. Time to CNS event was defined as the interval between the primary diagnosis of WM and the diagnosis of BNS or HT with CNS involvement. The cumulative incidence of CNS events was estimated using the Nelson-Aalen cumulative hazard function, accounting for events occurring at the time of primary WM diagnosis. Individual patient trajectories were depicted using a swimmer plot for visualization. Overall survival (OS) was defined as the time from WM diagnosis to death from any cause or last follow-up. OS from CNS involvement was measured from the diagnosis of BNS or CNS-HT to death from any cause or the last follow-up. Time-to-event analyses were visualized using the Kaplan-Meier method. All statistical analyses were performed using Stata Statistical Software version 18.0 (College Station, TX: StataCorp LLC).

## Results

### Incidence of CNS events

**Fig. 1:**
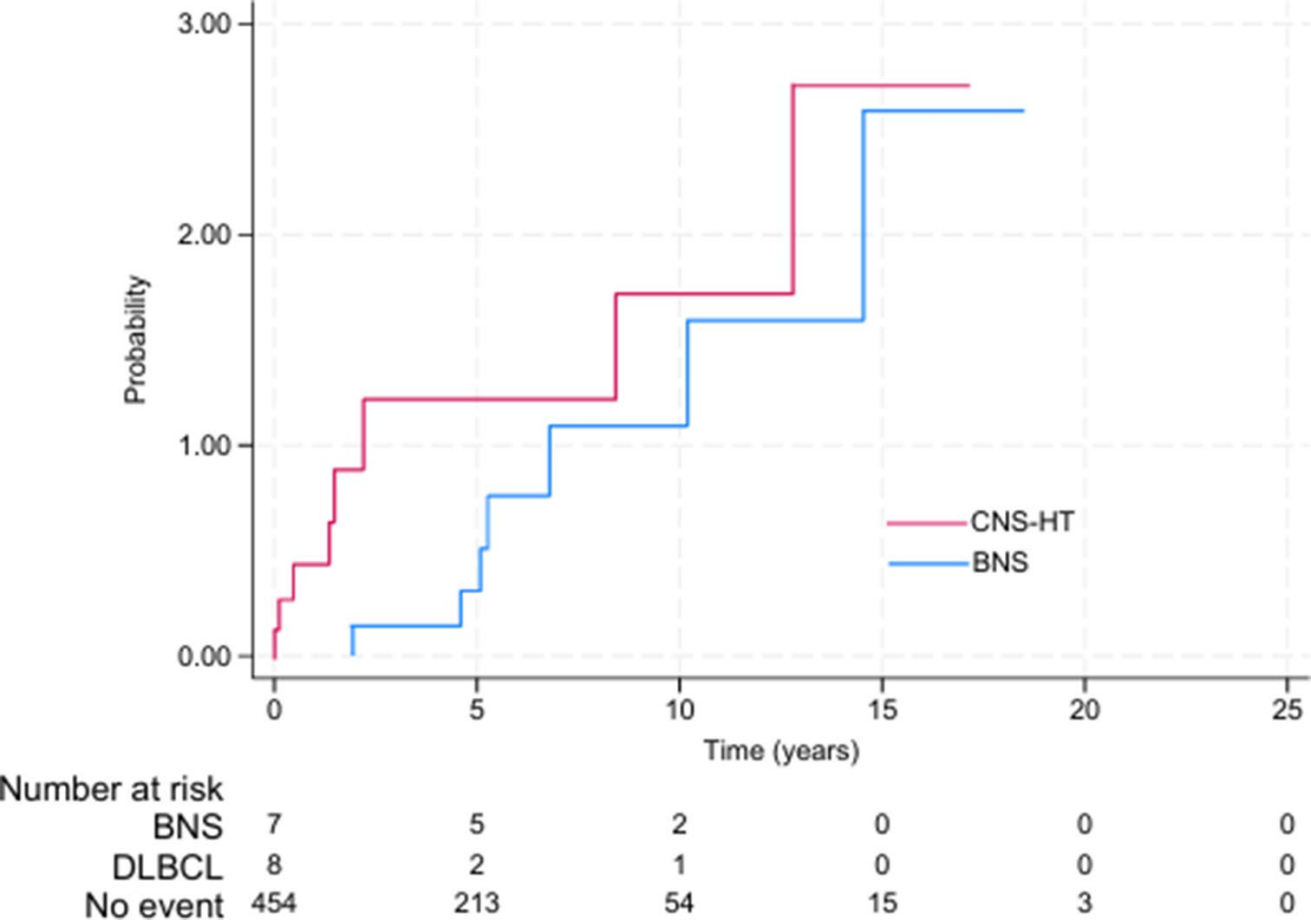
Cumulative risk of Bing-Neel syndrome (BNS) and histological transformation with central nervous system involvement (CNS-HT) from the time of Waldenström macroglobulinemia diagnosis

The total cohort consisted of 469 patients diagnosed with WM between 2000 and 2022. Morphologically proven BNS was identified in 7 (1.5%) patients, and CNS-HT was diagnosed in 8 out of 469 (1.7%) patients. The median time (range) from WM diagnosis to BNS diagnosis was 5.3 years (1.9–14.5), while the median time to CNS-HT diagnosis was 1.4 years (0-12.8). No cases of BNS were observed at the time of WM diagnosis, whereas 3 out of 8 (38%) patients with CNS-HT were diagnosed within the first six months following their primary WM diagnosis. The cumulative incidence of BNS and CNS-HT was 0.3% and 1.2% at 5 years, 1.1% and 1.7% at 10 years, and 2.6% and 2.7% at 15 years, respectively (Fig. 1).

### Characteristics of BNS

At the time of BNS diagnosis, the most common symptoms included cognitive impairment, motor or sensory disturbances, balance disorders, and visual disturbances. Notably, all patients experienced more than one CNS-related symptom. The characteristics of BNS patients at the onset of CNS involvement are summarized in Table 1. Four out of the 7 (57%) patients had MRI findings suggestive of CNS lymphoma. In addition to BNS, 3 (43%) patients presented with involvment of other extramedullary sites, including soft tissue and skeletal involvement.

**Table 1:** Clinical characteristics of patients with Bing-Neel syndrome (BNS) and histological transformation (HT) involving the central nervous system (CNS) at the time of CNS involvement

Variable	BNS (*n* = 7)	CNS-HT (*n* = 8)
Age, median (range), years	71 (65–77)	73 (66–85)
Hb, median (range), g/dL	11.8 (5.4–11.6)	12.1 (10.0-15.8)
Missing	1	0
IgM, median (range), g/L	5,4 (1.0–30.0)	7,1 (4.0–46.0)
Missing	1	0
Diagnostic sample, n (%):		
Parenchymal	2 (29)	4 (50)
CSF	5 (71)	2 (25)
Other extramedullary	0	2 (25)
MRI findings, n (%):		
Parenchymal	1 (14)	6 (75)
Leptomeningeal	2 (29)	0
Both	1 (14)	2 (25)
None	3 (43)	0
Extramedullary sites, n (%):		
Isolated CNS	4 (57)	4 (50)
Other extramedullary sites	3 (43)	4 (50)
Lines of therapy before CNS event, n (%):		
≥1	7 (100)	3 (37)
0	0 (0)	5 (63)
*MYD88*^L265P^ mutation, n (%):		
Bone marrow	5 (100)	8 (100)
Missing	2	0
CNS	1 (100)	7 (100)
Missing	6	1

*Abbreviations*: Hb: hemoglobin, IgM: immunoglobulin M, CSF: cerebrospinal fluid, MRI: magnetic resonance imaging

In all cases, the clonal B-cells detected in the CSF or brain biopsy exhibited an immunophenotypic profile comparable to that of the WM cells in the BM. The MYD88 L265P mutation was detected in all available primary diagnostic BM samples (5/5). Analysis for MYD88 L265P was only available from the CNS component in one patient in which it was detected.

**Fig. 2:**
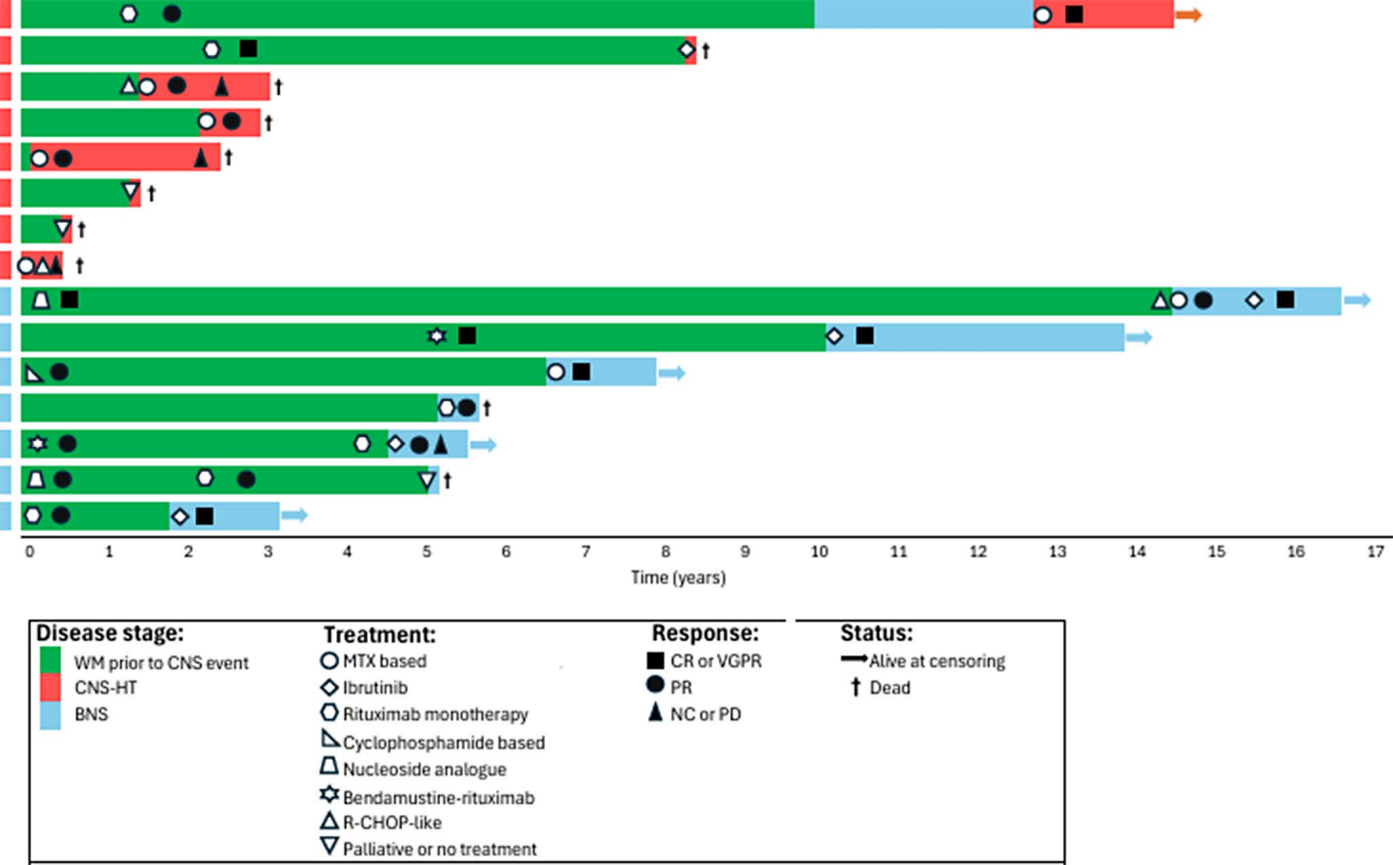
Swimmer plot illustrating the clinical course of patients with Waldenström macroglobulinemia and CNS involvement

The median number (range) of treatment lines administered prior to BNS diagnosis was 2 [1–3], with all patients receiving at least one treatment. Four patients received ibrutinib as first-line therapy for BNS, one patient was treated with high-dose methotrexate (HD-MTX)-based treatment, one patient received rituximab monotherapy, and one patient only received palliative therapy. All patients treated with ibrutinib had lasting responses, with three patients achieving complete remission (CR) of BNS-related manifestations. Figure 2 depicts the clinical trajectories of individual patients with CNS involvement, highlighting patterns of progression, timing of CNS events, treatment modalities, therapeutic responses, and survival outcomes.

### Characteristics of CNS-HT

The most common CNS symptoms at CNS-HT diagnosis were motor and sensory disturbances, paresis, cognitive impairment, cranial nerve deficits, and visual disturbances. Most patients presented with multiple symptoms. The clinical features of patients with CNS-HT are presented in Table 1. All patients had parenchymal lesions identified by MRI, with two (25%) patients displaying concurrent leptomeningeal involvement. Isolated CNS involvement was seen in 50% of patients with CNS-HT while the remaining 50% of patients had simultaneous involvement of multiple other extramedullary sites, including the kidneys, adrenal glands, testes, soft tissues and skeletal system. Notably, 6 (75%) patients had CNS involvement at the time of transformation, while it occurred at relapse in two (25%) patients.

The median (range) number of treatment lines administered prior to CNS-HT diagnosis was 0 (0–1). First-line treatments for CNS-HT were HD-MTX in three patients and sequential HD-MTX or intrathecal MTX combined with R-CHOP in two patients. One patient was treated with ibrutinib, while two patients received only palliative care without CNS-directed therapy. Due to advanced age and comorbidities, no patients underwent high-dose chemotherapy with autologous stem cell transplantation. Among the patients treated for HT, one achieved a CR and another a PR, whereas the remaining patients experienced refractory or early progressive disease. The clinical trajectories of patients with CNS-HT, including treatment strategies and responses, are illustrated in Fig. 2.

One patient with CNS-HT experienced a pre-phase of BNS before being diagnosed with transformation. Clonal MYD88 L265P mutated B-cells, consistent with LPL, were detected in both a vitrectomy and a retinal biopsy; three years later, the patient developed DLBCL, confirmed by a cerebellar biopsy. In another patient with CNS-HT, clonal B-cells without evidence of transformation were detected in the CSF, suggesting BNS. However, a subsequent biopsy revealed tumor infiltration of DLBCL in the brain parenchyma.

COO classification was available for 7 out of 8 patients with CNS-HT. One (14%) patient had a GC phenotype, and 6 (86%) had a non-GC phenotype. FISH analysis revealed no cases of MYC and BCL2 or MYC and BCL6 rearrangements; however, an isolated MYC gene rearrangement was detected in 1 (14%) patient. Ki67 expression ranged from 50 to 100%. The MYD88 L265P mutation was detected in all available samples, which included 8 WM BM samples and 7 DLBCL biopsies.

### Survival

**Fig. 3:**
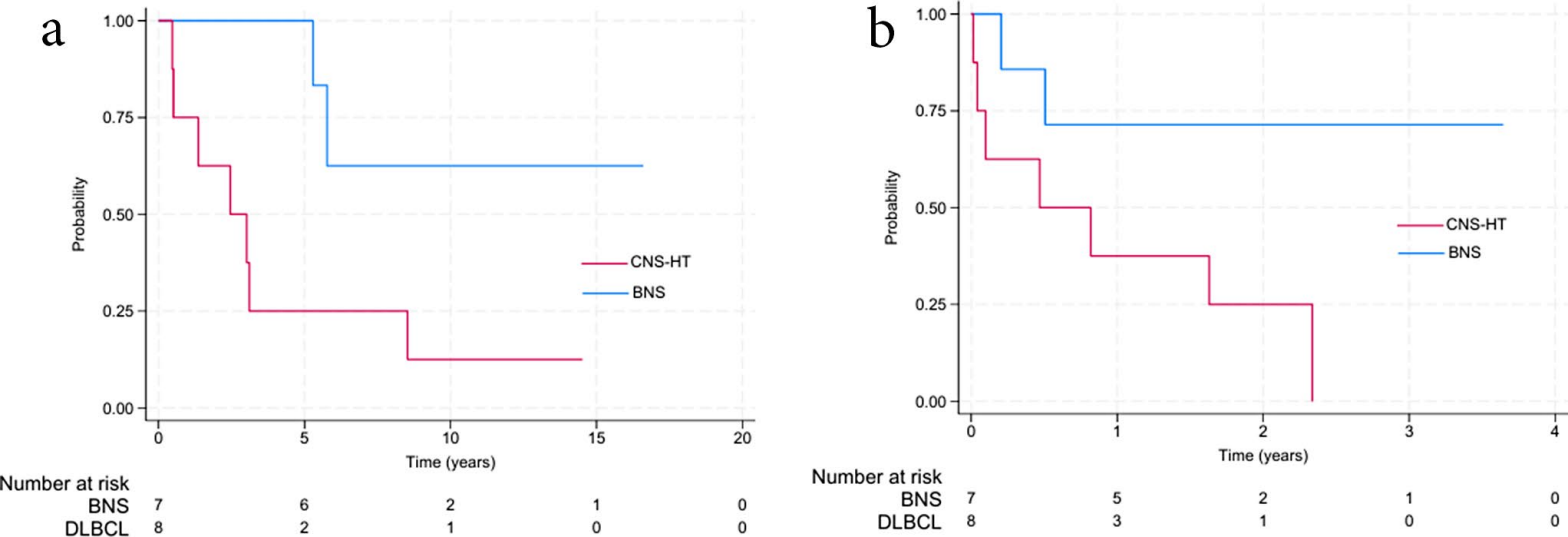
Overall survival in patients with Bing-Neel syndrome (BNS) and histological transformation with central nervous system involvement (CNS-HT) from primary Waldenström macroglobulinemia diagnosis (**a**) and from diagnosis of CNS involvement (**b**)

The median (range) follow-up time from WM diagnosis was 5.3 years (0.5–16.6). Kaplan-Meier estimates of OS from WM diagnosis for patients with BNS and CNS-HT are shown in Fig. 3. The median OS for patients with CNS-HT was 3.0 years (95% CI: 0.5–3.1). For patients with BNS, the median OS had not yet been reached at the time of follow-up (Fig. 3a). The median OS after CNS involvement was 9.8 months (95% CI: 0.5–19.6) for patients with CNS-HT, while the median OS for patients with BNS had not been reached (Fig. 3b). At the time of follow-up, two deaths unrelated to lymphoma were recorded among patients with BNS. In patients with CNS-HT, the causes of death included lymphoma progression in six patients and non-lymphoma-related causes in one patient.

## Discussion

This study reports on CNS involvement in WM in an unselected, population-based cohort diagnosed consecutively over two decades. To our knowledge, it is the first to concurrently present data on both BNS and CNS-HT, showing that these conditions occur at similar frequencies (1.5% for BNS and 1.7% for CNS-HT) and have comparable cumulative incidences over a 15-year period (2.6% and 2.7%, respectively). Previous studies have reported varying incidence rates for BNS (1–5%) and HT (1–4%), identifying the CNS as one of the most common sites of transformation [7, 8, 16, 17]. The median time from the diagnosis of WM to BNS has been reported between 3 and 6 years, with rare cases of BNS as the initial disease manifestation [6, 7]. Our results were comparable; however, BNS occurred only in previously treated patients, and no cases were detected within the first year following primary WM diagnosis. In contrast, CNS-HT occurred earlier, suggesting that early CNS events are more likely to represent transformation rather than BNS. Consistent with this, a previous study also found that a significant portion of transformed WM patients presented with transformation already at the time of WM diagnosis [11].

Distinguishing BNS from CNS-HT can be challenging in a clinical setting, highlighting the importance of an accurate diagnostic evaluation to improve early detection rates and prevent CNS injury, as recommended in consensus guidelines [5, 6]. In our study, patients with CNS involvement presented with a diverse range of CNS-related symptoms that did not reliably discriminate between BNS and CNS-HT. Furthermore, both conditions were also observed in patients without concurrent signs of systemic disease progression. However, we identified some clinical patterns, including generally less pronounced neurological deficits in patients with BNS. All patients with CNS-HT displayed parenchymal lesions, whereas imaging findings in BNS were less consistent, with nearly half of these patients exhibiting no detectable changes on MRI. Previous studies found MRI abnormalities in about 80% of patients with BNS, with leptomeningeal involvement being the most common finding, whereas tumoral masses were only observed in around 20% [6, 18, 19]. In CNS-HT, parenchymal or leptomeningeal involvement alone or in combination has been found in approximately 95% of cases [12]. Our findings suggest that CNS-HT is more likely in cases with early-onset neurological symptoms following the primary WM diagnosis and in treatment-naïve patients. This underscores the importance of thorough evaluation for potential disease transformation in patients with rapidly emerging manifestations. Given the overlapping clinical presentations, diagnostic clarification should prioritize CSF analysis, complemented by parenchymal biopsy when technically feasible and when risks are acceptable, especially in patients exhibiting parenchymal lesions. Notably, we also observed frequent involvement of other extramedullary sites outside the CNS in patients with both BNS and CNS-HT, suggesting that CNS involvement may often be part of a more diffuse disease dissemination. This highlights the value of systemic imaging, such as CT or positron emission tomography (PET) scans, to evaluate for extramedullary dissemination in patients with suspected CNS involvement. Additionally, biopsy of alternative extramedullary sites should be considered, particularly when transformation is suspected.

We also report a case in which BNS preceded high-grade transformation in the CNS by several years, which, to our knowledge, has not been previously documented. This underscores the importance of considering transformation in BNS patients who experience new or progressive neurological symptoms. In another patient, clonal B-cells were detected in the CSF with an immunophenotype consistent with LPL cells in the diagnostic BM, initially interpreted as BNS without transformation. However, histological examination of a subsequent brain biopsy was consistent with DLBCL, illustrating that cytological evaluation can carry inherent uncertainty in clearly distinguishing between LPL and DLBCL.

The MYD88 L265P mutation is found in approximately 95% of patients with WM and is also highly recurrent in BNS [6, 8, 10]. In line with previous reports, we identified MYD88 L265P mutations in the BM of all BNS patients tested. MYD88 wild-type has been reported as an independent predictor of transformation in WM [17, 20]. Nevertheless, MYD88 L265P remains a common feature in transformed WM and appears to increase the risk of CNS relapse [4, 12, 17]. This is consistent with studies showing strikingly high frequencies of MYD88 mutations in primary CNS lymphoma, as well as an increased risk of CNS recurrence in systemic de novo DLBCL patients carrying the MYD88 L265P mutation, particularly in the MCD genetic subtype [21, 22]. In our study, the MYD88 L265P mutation was detected in LPL cells from the BM of all patients with CNS-HT, and it was retained in DLBCL cells of all seven patients tested. The majority of MYD88 L265P mutated WM patients who experience transformation seemingly harbor the mutation in both neoplastic components, although molecular data are still scarce [23–25].

Most patients in our cohort with CNS-HT expressed a non-GC phenotype, and no cases of high-grade lymphoma were observed, which is in accordance with previous studies [4, 17, 23]. The non-GC subtype has been associated with the MYD88L265P mutation, extranodal disease, and poor outcome in de novo DLBCL, and these molecular and clinical traits appear to also characterize HT in patients with WM [4, 26]. Interestingly, Bruton’s tyrosine kinase (BTK) inhibitors have shown selective activity in MYD88L265P mutated DLBCL of the non-GC type, and numerous combination regimens currently are being tested in clinical trials [21]. Given the challenges of high-dose chemotherapy for elderly WM patients, targeted combination regimens present a promising, less toxic option to achieve more lasting responses.

Establishing the clonal relationship between DLBCL and WM is clinically important, as it distinguishes true transformation from de novo DLBCL; however, data on clonal origin remain limited [23, 24, 27]. Data on the clonal relationship were not available in our cohort, and studies exploring its clinical significance in patients with transformed WM are warranted.

Our study contributes to the limited survival data available on WM with CNS involvement. Retrospective studies on BNS have reported variable outcomes ranging from a 5-year survival of 71% to a median OS of only 2.4 years from the diagnosis of BNS [9, 16]. However, the diversity of initial therapies complicates comparisons across various studies. In BNS patients from our cohort, the prognosis was more favorable with a median survival that was not yet reached at follow-up with only two deaths recorded and both were not lymphoma related. Notably, four patients received ibrutinib as first line therapy for BNS, and all achieved a lasting response, which may partly explain the favorable prognosis. These results align with a previous case series on BNS patients treated with ibrutinib, which reported a 5-year BNS survival rate of 86% [28]. The outcome of patients with CNS-HT has only been reported in a single study with a prognosis comparable to other transformed WM patients, with a median survival of only 1 year following transformation [10]. In our study, the outcome following the onset of CNS-HT was equally poor, with a median survival of less than one year. None of the patients were candidates for consolidation with autologous stem cell transplantation. This further highlights the need for new treatment strategies for these patients.

The current study is limited by its retrospective design and the rarity of both BNS and CNS-HT. In some patients clinically suspected of CNS involvement, a definitive diagnosis may not be established due to the poor general condition of the patient, technical difficulties in obtaining a CNS biopsy, or an unacceptable risk of complications. Additionally, LPL/WM cells may seemingly also be present in CSF at a subclinical level that could be overlooked. Therefore, as in previous reports, the actual incidence of CNS involvement in WM might be underestimated due to insufficient diagnostic work-up. However, reviewing all patients in the current study, we identified only one patient with radiological findings suggestive of CNS lymphoma without subsequent diagnostic confirmation due to the patients’ general condition. The primary limitation of this study is its small sample size, reflecting the rarity of CNS events in WM patients. This low incidence presents significant challenges to prospective data collection. Consequently, large-scale studies encompassing broader populations are warranted to further elucidate these uncommon conditions.

In conclusion, CNS involvement in WM is rare, with BNS and CNS-HT occurring at similar rates in our real-world data. They present with heterogeneous clinical features and overlapping symptoms; however, early CNS involvement and parenchymal brain lesions were indicative of transformation. While the prognosis following CNS-HT was very poor, patients with BNS exhibited a less aggressive clinical course, responded better to treatment, and achieved more favorable outcomes. Lastly, the possibility of transformation should be considered in patients with BNS who experience new or progressive neurological symptoms.

## Data Availability

The data from this study can be made available upon reasonable request to the corresponding author and with the appropriate approvals from the Region Zealand Data Protection Agency (ID: REG-020-2022).
